# Identification of hemolytic anemia in Korean indigenous cattle with a criteria value of reticulocyte count, indirect bilirubin, and L-lactate concentration

**DOI:** 10.3389/fvets.2024.1375518

**Published:** 2024-08-21

**Authors:** Youngwoo Jung, Ji-Yeong Ku, Youngjun Kim, WooChan Kim, Hyungjae So, Lianfu Piao, Ji-Seon Yoon, Jinho Park

**Affiliations:** ^1^Department of Veterinary Internal Medicine, College of Veterinary Medicine, Jeonbuk National University, Iksan, Republic of Korea; ^2^Hanwoo (Korean Indigenous Cattle) Genetic Improvement Center, National Agricultural Cooperative Federation, Seosan, Republic of Korea

**Keywords:** bovine, bilirubin, Hanwoo (*Bos taurus* coreanae), hemolytic anemia, indirect bilirubin, Korean indigenous cattle, L-lactate, reticulocyte

## Abstract

Bovine hemolytic anemia has a negative impact on animal welfare and productivity due to its associated clinical symptoms. Hemolysis is generally known to cause reticulocytosis, increased indirect bilirubin, decreased concentration of haptoglobin, and increased lactate dehydrogenase. Additionally, tissue hypoperfusion due to concomitant anemia increases blood lactate concentration. However, few studies have reported the correlation between these indicators and hemolytic anemia in cattle. We expected that alterations in hematological and biochemical parameters could identify cattle with hemolytic anemia. Therefore, in addition to reporting differences in indicators according to hemolytic anemia, this study aimed to derive indicators and set criteria for identification of bovine hemolytic anemia. In cattle with hemolytic anemia, reticulocytosis, increased indirect bilirubin, and increased L-lactate were observed, and the correlation of these indicators with hematocrit (HCT) was confirmed. And since HCT alone has limitations in identifying hemolytic anemia, we suggest additional criteria to identify hemolytic anemia in cattle.

## 1 Introduction

Anemia is characterized by decreased red blood cell (RBC) counts, hemoglobin (Hb) concentrations, and/or hematocrit (HCT) levels (1). Anemia can cause clinical symptoms such as pallor, jaundice, weakness, lethargy, loss of appetite, and sometimes hemoglobinuria in cattle. Hypoxia accompanying anemia can result in systemic dysfunction (2–5). In addition, it is known to cause economic losses to the livestock industry through weight loss, reduced milk production, abortion, stillbirth, and, in severe cases, death (2, 3, 5–7).

Anemia can be classified into normocytic, macrocytic, and microcytic, depending on the size of the RBCs, and normochromic and hypochromic anemia, depending on the hemoglobin concentration. Depending on the bone marrow reaction, it can be classified into regenerative and non-regenerative anemia. The main cause of regenerative anemia is hemorrhage or hemolysis. Hemolysis involves the destruction of RBCs before they reach their normal lifespan, which in bovine is 130–160 days (1, 8).

Causes of hemolytic anemia in cattle include toxic plants such as cabbage (*Brassica* spp.), onion (*Allium cepa*), rye grass (*Lolium* spp.), or red maple (*Acer rubrum*). And tick-borne pathogens (*Ehrlichia* spp., *Anaplasma* spp., *Bartonella* spp., *Rickettsia* spp., *Theileria* spp., *Babesia* spp.) and other pathogens that infect RBCs (*Typanosoma* spp., *Sarcocystis* spp., *Mycoplasma* spp., *Leptospira* spp., *Clostridium* spp.), toxicosis (arsenic, copper, lead) or deficiency (copper, phosphorus) of certain elements can also cause hemolytic anemia in cattle. Primary or idiopathic immune-mediated hemolytic anemia has been reported rarely (8–11). Grazing on pastures, in particular, is known to have benefits in improving animal health, animal welfare, and farm productivity. Consequently, the number of cattle grazing on pastures is increasing in Korea, and with the climate in Korea transitioning to warmer conditions, there is a rise in cases of infection caused by tick-borne pathogens that can induce hemolytic anemia in Korean indigenous cattle (Hanwoo) (10, 12). In other words, the risk of exposure to tick-borne pathogens is increasing, and the likelihood of developing hemolytic anemia as a result of subsequent diseases is expected to be high. However, most previous studies have focused on detecting pathogens that cause hemolytic anemia (13, 14) or alterations in the hematological and biochemical parameters associated with each pathogen infection (15–17). A study on grazing Hanwoo reported a significantly higher prevalence of *Theileria orientalis* infections compared to housing cattle, and these grazing cattle showed markedly lower RBC counts and HCT (12, 18). Similarly, it was shown that *Theileria orientalis* infection rates increase during the summer, leading to significant changes in the RBC profiles of grazing cattle (12, 19).

Hemolysis is generally known to result in reticulocytosis, increased indirect bilirubin, decreased haptoglobin, and increased lactate dehydrogenase (1, 8, 20). However little research has been reported on the correlation between such indicators and hematological parameters in bovine hemolytic anemia. No studies have reported a correlation between L-lactate levels, which are closely related to hypoxia (21, 22), and bovine hemolytic anemia.

Clinical identification of hemolytic anemia is necessary and important to screen sick animals to determine appropriate diagnosis and treatment, and to understand the impact on productivity. We predicted that the more severe the hemolysis, the greater the changes in hematological and biochemical parameters, and that this would enable to identify cattle with hemolytic anemia. The main objective of this study was to evaluate the correlation between hematological parameters and additional indicators that can be used to identify hemolytic anemia. Furthermore, we suggest additional criteria for identifying hemolytic anemia by correlating the degree of anemia with the degree of change in the values of some indicators.

## 2 Materials and methods

### 2.1 Animals

During the month of June 2023, 75 cattle suspected of anemia due to clinical symptoms such as pallor, anorexia, weakness, and lethargy were included in the study at a farm raising more than about 500 cattle in Chungcheongnam-do, South Korea. Among these 75 cattle, 15 were housed in barns, while the remaining 60 were grazing in pastures. All the cattle were female Korean indigenous cow aged between 22 and 46 months. These cattle did not show major clinical sign such as diarrhea or respiratory symptoms, and there was no clinical evidence of bleeding. This study was approved by the Institutional Animal Care and Use Committee of the National Institute of Animal Science, Republic of Korea (JBNU IACUC No. NON2023-123).

### 2.2 Blood sample

Blood samples were taken from the jugular vein of cattle and collected into 3 mL EDTA tubes (BD Vacutainer, Beckton Dickinson Vacutainer Systems, Franklin Lakes, NJ, United States) and 5 mL serum separating tubes (Vacutte serum tube, Greiner Bio-One, Austria). Blood collection was performed over multiple days and took 2–3 h each day. The tubes were transported to the laboratory in a refrigerated state, and delivery was completed within 1 h. The serum separating tubes were left standing for at least 1 h and then the serum was separated by centrifugation at 3,000 × g for 10 min. All whole blood collected in EDTA tubes was used for testing on the day of blood collection. Serum that could not be tested on the same day was stored frozen (−24°C) and used for testing within 3 days.

### 2.3 Measurement of hemolytic anemia parameters

At the farm, native whole blood was used for lactate measurement immediately before being collected into blood tubes using a portable lactate device (Nova Biomedical, Waltham, MA, United States). It is based on an enzymatic amperometric system and what is measured is L-lactate (23, 24). In the laboratory, EDTA whole blood was used for complete blood count. The complete blood count was performed on the day of blood collection and was performed using an automated blood cell analyzer (IDEXX ProCyte Dx; IDEXX Laboratories, Westbrook, ME, United States) (25). Blood smear was not performed due to time constraints. Bilirubin and haptoglobin were measured using separated serum. The total bilirubin and direct bilirubin concentrations were measured using an automated dry biochemical analyzer (FUJI DRI-CHEM 4000i, Fujifilm, Tokyo, Japan) as in previous studies (26, 27). This device measures bilirubin using the diazo reaction. Direct bilirubin reacts with the diazonium salt of benzenesulfonic acid to form a diazo dye. Indirect bilirubin dissociates with dyphylline and undergoes diazo reaction together with direct bilirubin to form diazo dye. The indirect bilirubin concentration was determined by subtracting the direct bilirubin concentration from the total bilirubin concentration (28). Haptoglobin concentrations were measured using a commercial colorimetric assay kit (Phase Colorimetric Assay, Tridelta Development Ltd., Maynooth, Ireland), following the manufacturer’s instructions. This used the haptoglobin-hemoglobin binding method (29).

### 2.4 Statistics

Statistical analyses of the blood test results were performed using the SPSS 29.0 software package (SPSS, Chicago, IL, United States). Normality test of the data was performed using the Kolmogorov–Smirnova method. One-way ANOVA and Kruskal–Wallis test were used to compare blood test results between groups according to the severity of anemia. In comparing anemic and criteria-normal group, statistical significance was confirmed using *t*-test and Mann–Whitney U test. Data were described as mean ± standard deviation (SD), and statistically significant differences were determined at *p* < 0.05. In addition, Pearson’s correlation test was performed to confirm the correlation between parameters, and Pearson’s correlation coefficient was *r* ≥ 0.7, strong correlation; 0.5 ≤ *r* < 0.7, moderate correlation; 0.3 ≤ *r* < 0.5, weak correlation; and *r* < 0.3, no correlation (30).

## 3 Results

### 3.1 Differences in hematological and hemolytic parameters according to anemia severity

**Figure 1:**
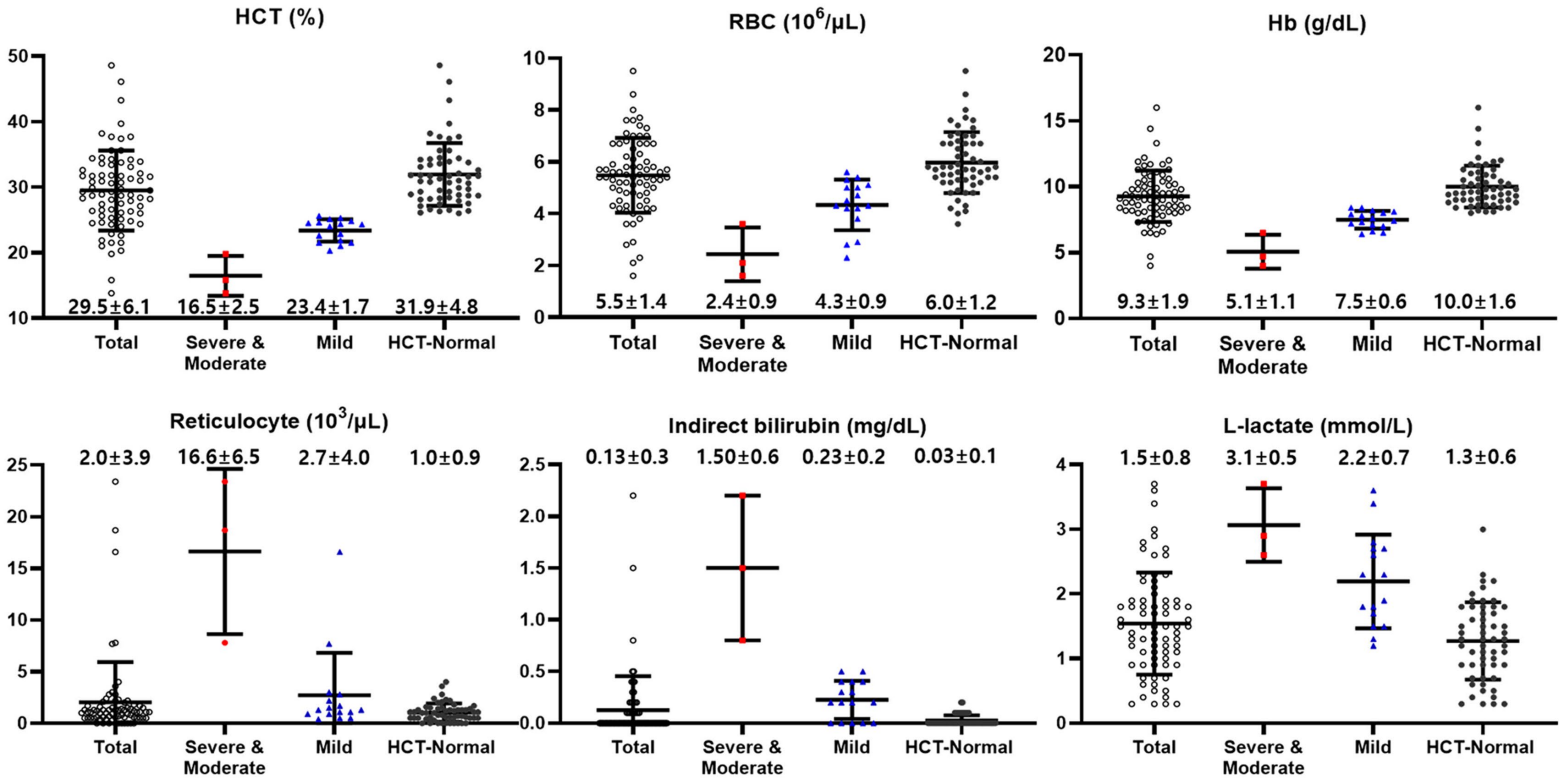
Hematological and biochemical values of cattle classified according to anemia severity based on the HCT levels [Normal (≥26%), Mild (20–26%), and Severe to moderate (<20%)]. Total (black empty circle): total cattle (*n* = 75); HCT-Normal (red square): HCT-normal cattle (*n* = 56); Mild (blue triangle): mild anemic cattle (*n* = 16); Severe to Moderate (black circle): severe to moderate anemic cattle (*n* = 3).

Cattle were classified according to anemia severity based on the HCT level. Hematocrit less than 20% was determined to be the severe to moderate group, HCT 20–26% was the mild group, and HCT 26% or more was determined to be the HCT-normal group (31). The severe to moderate group included three cattle, the mild group included 16 cattle, and the HCT-normal group consisted of 56 cattle. The hematological and hemolysis parameters for each group are depicted in (Figure 1). Their respective mean ± SD (95% Confidence Interval) HCT levels (%) were 16.5 ± 2.5 (10.3–22.7), 23.4 ± 1.7 (22.5–24.3), and 31.9 ± 4.8 (30.7–33.2). Red blood cell counts (10^6^/μL) were 2.4 ± 0.9 (0.3–4.6), 4.3 ± 0.9 (3.8–4.8), and 6.0 ± 1.2 (5.6–6.3), respectively, showing statistically significant differences (*p* < 0.01). Hemoglobin concentrations (g/dL) were 5.1 ± 1.1 (2.5–7.7), 7.5 ± 0.6 (7.2–7.8), and 10.0 ± 1.6 (9.6–10.4), respectively, with statistically significant differences observed (*p* < 0.01). However, no significant differences were observed in the mean corpuscular volume (MCV) (fL) and mean corpuscular hemoglobin concentration (MCHC) (g/dL) values among the groups.

Reticulocyte counts (10^3^/μL) were 16.6 ± 6.5 (0.4–32.9) for the severe to moderate group, 2.7 ± 4.0 (0.6–4.8) for the mild group, and 1.0 ± 0.9 (0.8–1.3) for the HCT-normal group. Platelet counts (10^3^/μL) were 123 ± 50 (−2–247), 145 ± 65 (110–179), and 186 ± 70 (168–205), respectively, with statistically significant difference observed (*p* < 0.05).

Total bilirubin concentrations (mg/dL) were 1.7 ± 0.7 (0.08–3.32), 0.33 ± 0.2 (0.23–0.43), and 0.13 ± 0.1 (0.11–0.14), respectively (*p* < 0.01). Indirect bilirubin concentrations (mg/dL) were 1.50 ± 0.6 (0.08–2.92), 0.23 ± 0.2 (0.13–0.32), and 0.03 ± 0.1 (0.01–0.04) respectively, with a significant increase observed, especially in the severe to moderate group.

L-lactate concentrations (mmol/L) were 3.1 ± 0.5 (1.9–4.2), 2.2 ± 0.7 (1.8–2.6), and 1.3 ± 0.6 (1.1–1.4), respectively, and the highest was confirmed in the severe to moderate group (*p* < 0.01). Haptoglobin concentrations (mg/dL) were 18.0 ± 1.5 (14.3–21.7), 12.0 ± 3.4 (10.2–13.8), and 11.4 ± 3.8 (10.4–12.4), respectively.

### 3.2 Correlation between HCT and hemolytic parameters according to anemia severity

The correlation between HCT and hemolytic parameters in a total of 75 cattle confirmed a correlation with reticulocyte count (*r* = −0.467, *p* < 0.01), indirect bilirubin (*r* = −0.574, *p* < 0.01), and L-lactate (*r* = −0.646, *p* < 0.01) levels. No significant correlations were observed between MCV, MCHC, platelet count, and haptoglobin levels.

The correlation between HCT and hemolytic parameters was analyzed within each group, classified according to the severity of anemia. A strong correlation was found between HCT and reticulocyte count levels in the severe to moderate group (*r* = −0.998, *p* < 0.05). Additionally, significant correlations were observed between HCT and indirect bilirubin (*r* = −0.731, *p* < 0.01), as well as L-lactate (*r* = −0.722, *p* < 0.01) levels in the mild group.

### 3.3 Blood test results in cattle classified according to hemolytic parameters

Additional parameters were established to identify hemolytic anemia in cattle. Specifically, parameters strongly correlated with HCT level in anemic cattle, such as reticulocyte count, indirect bilirubin, and L-lactate concentration, were used as additional hemolytic parameters. If the upper limit of the 95% CI value (reticulocyte 1.3 × 10^3^/μL, indirect bilirubin 0.04 mg/dL, and L-lactate 1.4 mmol/L) of the hemolytic parameter of the HCT-normal group was exceeded, it was determined to be anemic cattle.

Of the 75 cattle, 25 had reticulocyte count exceeding 1.3 × 10^3^/μL, with 13 of these in the HCT-normal group. Twenty-six cattle had indirect bilirubin concentration exceeding 0.04 mg/dL, with 12 of them in the HCT-normal group. Thirty-six cattle had L-lactate concentration exceeding 1.4 mmol/L, among which six were in the HCT-normal group. In total, 26 cattle with normal HCT were additionally determined to have hemolytic anemia based on hemolytic parameters.

Differences in hematological and hemolytic parameters of each group classified according to reticulocyte count, indirect bilirubin or L-lactate levels are shown in Table 1. Hematocrit of anemic cattle was 22.3 ± 3.1 based on HCT, 28.7 ± 8.0 based on reticulocyte, 26.0 ± 6.9 based on indirect bilirubin, and 26.2 ± 4.9 based on L-lactate. Reticulocyte was lower in anemic cattle based on L-lactate (3.0 ± 5.1) compared to anemic cattle based on HCT (4.9 ± 6.8), and it was also lower than anemic cattle based on reticulocyte (4.7 ± 5.8) and indirect bilirubin (3.8 ± 6.1). Total bilirubin was lower in anemic cattle based on L-lactate (0.31 ± 0.5) and reticulocyte (0.36 ± 0.6) compared to that based on HCT (0.55 ± 0.6). In addition, indirect bilirubin was lower in anemic cattle based on L-lactate (0.20 ± 0.4) and reticulocyte (0.24 ± 0.5) compared to anemic cattle based on HCT (0.43 ± 0.5). The lowest L-lactate was observed in anemic cattle based on reticulocyte (1.8 ± 0.7), compared to that based on HCT (2.3 ± 0.7), indirect bilirubin (1.9 ± 0.9) and L-lactate (2.1 ± 0.6) but there was no statistically significant difference among groups.

**Table 1:** Blood test results of cattle classified based on reticulocyte, indirect bilirubin, and L-lactate.

	Classified using reticulocyte		Classified using indirect bilirubin		Classified using L-lactate	
	Reticulocyte-normal (*n* = 50)	Anemia (*n* = 25)	Indirect bilirubin-normal (*n* = 49)	Anemia (*n* = 26)	L-lactate-normal (*n* = 36)	Anemia (*n* = 39)
HCT (%)	29.9 ± 4.8 (28.5–31.3)	28.7 ± 8.0 (25.4–32.0)	31.4 ± 4.6 (30.1–32.7)	26.0 ± 6.9 (23.2–28.7)^**^	33.1 ± 5.2 (31.3–34.8)	26.2 ± 4.9 (24.6–27.8)^**^
RBC (10⁶/μL)	5.7 ± 1.2 (5.3–6.0)	5.1 ± 1.8 (4.4–5.9)	5.9 ± 1.0 (5.6–6.2)	4.6 ± 1.7 (3.9–5.3)^**^	6.1 ± 1.3 (5.7–6.5)	4.9 ± 1.4 (4.5–5.4)^**^
Hb (g/dL)	9.4 ± 1.5 (9.0–9.9)	8.9 ± 2.5 (7.9–10.0)	9.9 ± 1.4 (9.5–10.3)	8.1 ± 2.2 (7.2–9.0)^**^	10.3 ± 1.7 (9.8–10.9)	8.3 ± 1.5 (7.8–8.8)^**^
MCV (fL)	53.8 ± 7.4 (52–56)	59.9 ± 14.0 (54–66)	53.6 ± 6.5 (52–55)	60.0 ± 14.6 (54–66)	55.5 ± 7.8 (53–58)	56.1 ± 12.5 (52–60)
MCHC (g/dL)	31.6 ± 1.4 (31–32)	31.1 ± 1.8 (30–32)	31.6 ± 1.4 (31–32)	31.1 ± 1.7 (30–32)	31.3 ± 1.5 (31–32)	31.6 ± 1.5 (31–32)
Reti (10^3^/μL)	0.7 ± 0.4 (0.6–0.8)	4.7 ± 5.8 (2.3–7.1)^**^	1.1 ± 0.9 (0.8–1.3)	3.8 ± 6.1 (1.3–6.2)	0.9 ± 0.7 (0.7–1.2)	3.0 ± 5.1 (1.4–4.7)^*^
Pla (10^3^/μL)	177 ± 76 (156–199)	170 ± 60 (146–195)	181 ± 66 (162–200)	164 ± 78 (132–196)	174 ± 72 (150–198)	176 ± 70 (153–198)
To–Bil (mg/dL)	0.17 ± 0.1 (0.13–0.21)	0.36 ± 0.6 (0.13–0.59)	0.10 ± 0.0 (0.10–0.10)	0.48 ± 0.5 (0.27–0.69)^**^	0.15 ± 0.1 (0.11–0.19)	0.31 ± 0.5 (0.16–0.46)
Di–Bil (mg/dL)	0.10 ± 0.0 (0.10–0.11)	0.11 ± 0.0 (0.09–0.13)	0.10 ± 0.0 (0.10–0.10)	0.12 ± 0.0 (0.10–0.13)^*^	0.10 ± 0.0 (0.10–0.11)	0.11 ± 0.0 (0.10–0.12)
In–Bil (mg/dL)	0.07 ± 0.1 (0.03–0.11)	0.24 ± 0.5 (0.03–0.46)	0.00 ± 0.0 (0.00–0.00)	0.37 ± 0.5 (0.18–0.56)^**^	0.05 ± 0.1 (0.01–0.08)	0.20 ± 0.4 (0.06–0.34)
L-lac (mmol/L)	1.4 ± 0.8 (1.2–1.6)	1.8 ± 0.7 (1.5–2.1)^*^	1.4 ± 0.6 (1.2–1.6)	1.9 ± 0.9 (1.5–2.2)^*^	0.9 ± 0.4 (0.8–1.0)	2.1 ± 0.6 (1.9–2.3)^**^
Hap (mg/dL)	12.4 ± 3.6 (11.3–13.4)	10.7 ± 4.1 (9.0–12.4)	11.8 ± 3.8 (10.6–12.9)	11.9 ± 4.0 (10.3–13.5)	11.6 ± 4.2 (10.2–13.1)	11.9 ± 3.6 (10.8–13.1)

RBC, red blood cells; HCT, hematocrit; Hb, hemoglobin; MCV, mean corpuscular volume; MCHC, mean corpuscular hemoglobin concentration; Reti, reticulocytes; Pla: Platelets; To-Bil, total bilirubin; Di-Bil, direct bilirubin; In-Bil, indirect bilirubin; Hap, haptoglobin.

Mean ± SD (95% Confidence Interval).

^*^p < 0.05, ^**^p < 0.01.

### 3.4 Correlation between HCT and hemolytic parameters in anemic cattle classified according to HCT or hemolytic parameters

**Figure 2:**
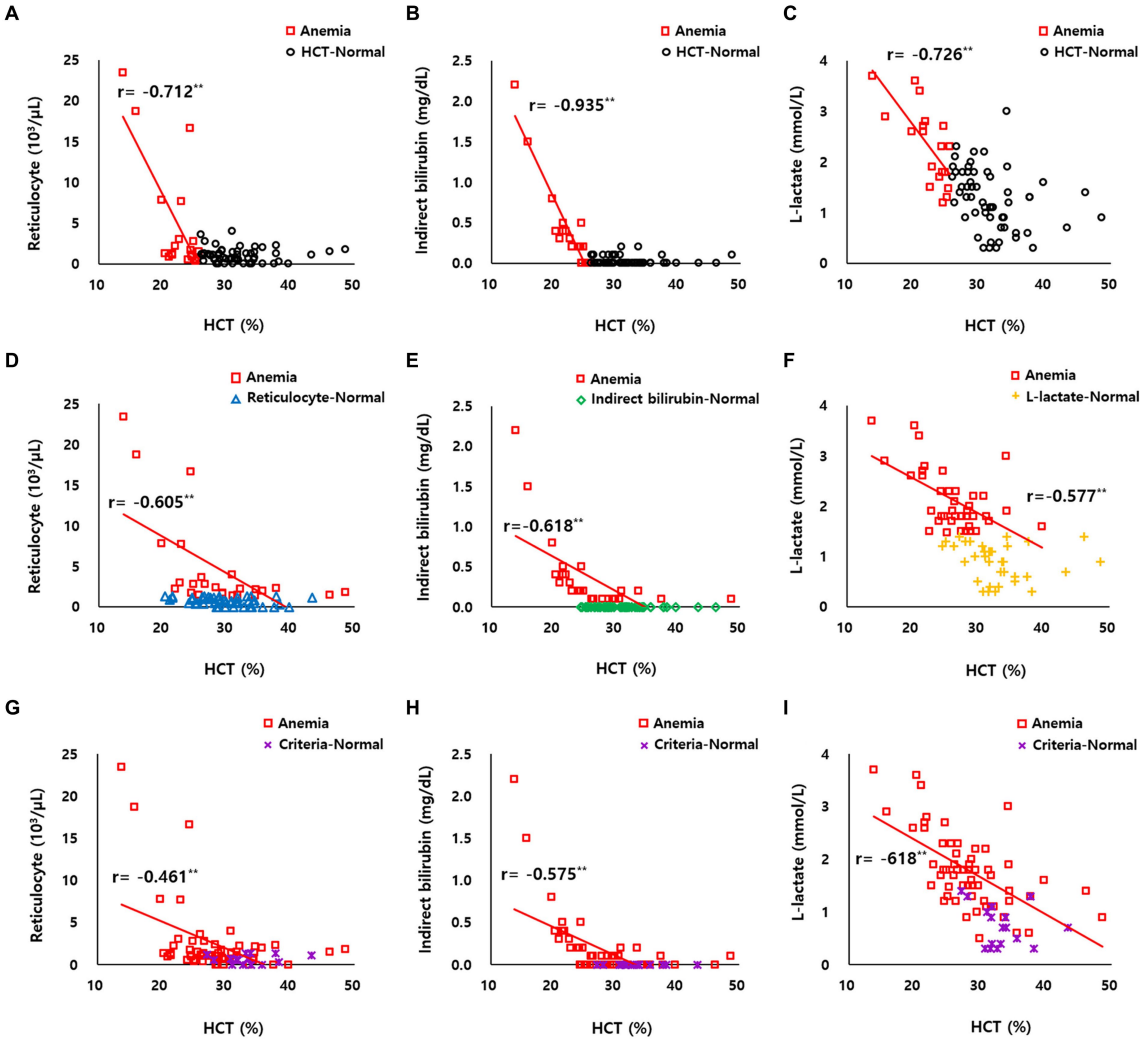
**(A–C)** Correlation between HCT and reticulocyte count, indirect bilirubin, and L-lactate levels of HCT-normal cattle (black circle dot, *n* = 56) and anemic cattle (red square dot, *n* = 19) based on HCT. **(D)** Correlation between HCT and reticulocyte count of reticulocyte-normal cattle (blue triangle dot, *n* = 56) and anemic cattle (red square dot, *n* = 19) based on reticulocyte count. **(E)** Correlation between HCT and indirect bilirubin of indirect bilirubin-normal cattle (green diamond dot, *n* = 56) and anemic cattle (red square dot, *n* = 19) based on indirect bilirubin. **(F)** Correlation between HCT and L-lactate of L-lactate-normal cattle (yellow plus-shaped dot, *n* = 56) and anemic cattle (red square dot, *n* = 19) based on L-lactate. **(G–I)** Correlation between HCT and reticulocyte count, indirect bilirubin, and L-lactate levels of criteria-normal cattle (purple x-mark dot, *n* = 56) and anemic cattle (red square dot, *n* = 19) based on on combined criteria of HCT, reticulocyte count, indirect bilirubin, and L-lactate levels. **p < 0.01.

The correlation between HCT and reticulocyte count was confirmed as *r* = −0.712 (*p* < 0.01) based on HCT (Figure 2A) and *r* = −0.605 (*p* < 0.01) based on reticulocyte (Figure 2D). The correlation between HCT and indirect bilirubin was *r* = −0.935 (*p* < 0.01) based on HCT (Figure 2B) and *r* = −0.618 (*p* < 0.01) based on indirect bilirubin (Figure 2E). The correlation between HCT and L-lactate was *r* = −0.726 (*p* < 0.01) based on HCT (Figure 2C) and *r* = −0.577 (*p* < 0.01) based on L-lactate (Figure 2F).

### 3.5 Blood test results in cattle classified according to criteria combined HCT and hemolytic parameters

When anemia was determined solely based on HCT, only 19 out of 75 cattle were classified as anemia. In contrast, when considering the combined criteria of HCT, reticulocyte count, indirect bilirubin, and L-lactate levels, 58 out of 75 cattle were identified as anemia (Table 2). Comparing the differences between anemic cattle and criteria-normal cattle, HCT was 28.4 ± 6.2 and 33.3 ± 3.7, RBC counts were 5.2 ± 1.4 and 6.3 ± 1.1, and Hb concentrations were 8.9 ± 1.9 and 10.6 ± 1.2, respectively, and there was a significant decrease in anemic cattle (*p* < 0.01). Additionally, anemic cattle showed a significant increase in reticulocyte count compared to criteria-normal cattle, with values of 2.4 ± 4.3 and 0.6 ± 0.5, respectively (*p* < 0.01). The platelet counts were 171 ± 67 and 188 ± 81, respectively, and was not significantly different.

**Table 2:** Blood test results of cattle classified based on HCT alone and combined hemolytic parameters.

	Classified using only HCT		Classified using HCT + 3 parameters^a^	
	HCT-normal (*n* = 56)	Anemia (*n* = 19)	Criteria-normal^b^ (*n* = 17)	Anemia (*n* = 58)
HCT (%)	31.9 ± 4.8 (30.7–33.2)	22.3 ± 3.1 (20.8–23.8)^**^	33.3 ± 3.7 (31.3–35.2)	28.4 ± 6.2 (26.8–30.0)^**^
RBC (10^6^/μL)	6.0 ± 1.2 (5.6–6.3)	4.0 ± 1.2 (3.5–4.6)^**^	6.3 ± 1.1 (5.7–6.8)	5.2 ± 1.4 (4.9–5.6)^**^
Hb (g/dL)	10.0 ± 1.6 (9.6–10.4)	7.1 ± 1.1 (6.6–7.7)^**^	10.6 ± 1.2 (10.0–11.3)	8.9 ± 1.9 (8.4–9.4)^**^
MCV (fL)	54.6 ± 7.8 (53–57)	59.3 ± 15.6 (52–67)	56.4 ± 11.3 (53–59)	53.8 ± 6.9 (50–57)
MCHC (g/dL)	31.3 ± 1.5 (31–32)	31.8 ± 1.7 (31–33)	31.3 ± 1.6 (31–32)	32.0 ± 1.3 (31–33)
Reticulocyte (10^3^/μL)	1.0 ± 0.9 (0.8–1.3)	4.9 ± 6.8 (1.7–8.2)^**^	0.6 ± 0.5 (0.4–0.9)	2.4 ± 4.3 (1.3–3.6)^**^
Platelet (10^3^/μL)	186 ± 70 (168–205)	141 ± 63 (111–172)^*^	188 ± 81 (146–230)	171 ± 67 (154–189)
Total bilirubin (mg/dL)	0.13 ± 0.1 (0.11–0.14)	0.55 ± 0.6 (0.26–0.83)^**^	0.10 ± 0.0 (0.10–0.10)	0.27 ± 0.4 (0.17–0.37)^**^
Direct bilirubin (mg/dL)	0.10 ± 0.0 (0.10–0.10)	0.12 ± 0.1 (0.10–0.15)^**^	0.10 ± 0.0 (0.10–0.10)	0.11 ± 0.0 (0.10–0.12)
Indirect bilirubin (mg/dL)	0.03 ± 0.1 (0.01–0.04)	0.43 ± 0.5 (0.16–0.69)^**^	0.00 ± 0.0 (0.00–0.00)	0.16 ± 0.4 (0.07–0.26)^**^
L-lactate (mmol/L)	1.3 ± 0.6 (1.1–1.4)	2.3 ± 0.7 (2.0–2.7)^**^	0.7 ± 0.4 (0.5–0.9)	1.8 ± 0.7 (1.6–2.0)^**^
Haptoglobin (mg/dL)	11.4 ± 3.8 (10.4–12.4)	13.0 ± 3.9 (11.1–14.8)	13.9 ± 4.2 (11.5–16.2)	11.3 ± 3.6 (10.3–12.2)^*^

a Reticulocytes, indirect bilirubin, and L-lactate.

b Cattle with HCT ≥ 26%, reticulocyte count < 1.3 × 10^3^/μL, indirect bilirubin < 0.07 mg/dL, and L-lactate < 1.6 mmol/L.

Mean ± SD (95% Confidence Interval).

*p < 0.05, **p < 0.01.

In anemic cattle, total bilirubin concentrations were 0.27 ± 0.4, compared to 0.10 ± 0.0 in criteria-normal cattle. Indirect bilirubin concentrations were 0.16 ± 0.4 and 0.00 ± 0.0 in anemic and criteria-normal cattle, respectively, showing a significant increase in anemic cattle (*p* < 0.01). L-lactate concentration was higher in anemic cattle, with values of 1.8 ± 0.7 compared to 0.7 ± 0.4 in criteria-normal cattle (*p* < 0.01). Additionally, haptoglobin concentrations were 11.3 ± 3.6 in criteria-normal cattle and 13.9 ± 4.2 in anemic cattle (*p* < 0.05) (Table 2).

### 3.6 Correlation between HCT and hemolytic parameters of cattle classified according to criteria combined HCT and hemolytic parameters

In anemic cattle classified based on the combined criteria of HCT, reticulocyte count, indirect bilirubin, and L-lactate levels, the correlation between HCT and reticulocyte count was *r* = −0.461 (*p* < 0.01) (Figure 2G), between HCT and indirect bilirubin was *r* = −0.575 (*p* < 0.01) (Figure 2H), and between HCT and L-lactate was *r* = −0.618 (*p* < 0.01) (Figure 2I).

## 4 Discussion

In general, hemolytic anemia is regenerative, and the bone marrow responds to RBC loss by increasing RBC production, releasing reticulocytes into the bloodstream. Consequently, during regenerative anemia, the number of reticulocytes in the bloodstream increases. Reticulocytes of most species remain in the bone marrow for 2–3 days before release and ultimately mature in the peripheral blood or spleen. However, in healthy cattle, reticulocytes mature in the bone marrow and are released as mature RBCs, so reticulocytes cannot be observed in the blood, and in response to anemia, a moderate increase occurs (1, 8). Additionally, it has been reported that the normal range for bovine reticulocytes is 0 × 10^3^/μL (1, 31). However, our results showed an increase in the reticulocyte count in all groups. It is thought that even if the hematological standard is normal, there may be insidious anemia, which may be caused by infection with benign *Theileria* species such as *Theileria orientalis* (32). It can also be that an occult and subclinical form of infection from pathogens can cause bovine hemolytic anemia (33, 34). The severe to moderate groups showed a greater increase in the reticulocyte count compared to the mild group. The severe to moderate groups showed macrocytic anemia, whereas the mild group showed normocytic anemia. This may be related to an increase in the number of immature RBCs in the bone marrow in response to severe anemia.

In present results, differences in platelet counts were observed among the severity groups of anemia, with a decrease noted in the more severe anemia groups. Platelet counts could falsely increase due to fragmented RBCs or white blood cells, and false decreases could occur due to platelet aggregation. Platelet aggregation could occur after exposure to EDTA (1, 31). However, in this study, platelet counts were measured only using an automated cell counter, and blood smear tests to verify platelet counts were not performed. There may be discrepancies between the reported results and the actual platelet counts.

Hemolysis is classified into intravascular and extravascular hemolysis depending on the site of occurrence. Intravascular hemolysis is the disruption of the RBC membrane in circulation, while extravascular hemolysis involves the phagocytosis of blood cells by macrophages, mainly in the spleen, with some occurring in the liver and bone marrow. During intravascular hemolysis, hemoglobin released from lysed RBCs forms a complex with haptoglobin. This complex is then metabolized into indirect bilirubin in hepatocytes and macrophages. Therefore, indirect bilirubin levels may increase, and haptoglobin levels may decrease. In contrast, hemoglobin is metabolized into indirect bilirubin in extravascular hemolysis and released in spleen macrophages. Haptoglobin levels may also decrease because it can be released from macrophages (1, 20, 35). In our study, an increase in indirect bilirubin levels was observed in the severe, moderate, and mild anemia groups, whereas it was barely detectable in the HCT-normal group. The increase in indirect bilirubin levels in the severe to moderate groups was greater than that in other groups. This shows that the more severe the hemolytic anemia, the more likely it is to be indirect bilirubinemia. However, the expected decrease in haptoglobin levels was not observed in any group; rather, the group with more severe anemia showed an increasing trend. In humans, haptoglobin can be used as an indicator of hemolysis (20), and in cattle, as an acute-phase protein, haptoglobin may be useful for diagnosing and monitoring inflammatory diseases (35–37). However, haptoglobin levels can be influenced by various factors, including inflammation and infection. Inflammation can trigger the release of cytokines, such as interleukin-6, that stimulate haptoglobin production, leading to increased haptoglobin levels as part of the acute-phase response (35, 36, 38, 39). Therefore, while haptoglobin can provide additional information in the diagnosis of hemolytic anemia, it has limitations as a specific indicator for diagnosing hemolytic anemia in cattle.

Lactate is an organic acid produced by the anaerobic metabolism of pyruvate during glycolysis. Almost all lactate produced by mammalian cells is considered L-lactate (1, 40). L-lactate is used as a clinical biomarker for disease severity and mortality in both human and veterinary medicine (41, 42). Hyperlactatemia is divided into two categories: Type A and Type B. Type A is the most common and the main causes are hypoxia and decreased tissue perfusion. Type B occurs when oxygen transport is sufficient but mitochondrial function or carbohydrate metabolism is altered. In veterinary medicine, common causes include underlying diseases such as diabetes mellitus, liver disease, neoplasms, and sepsis (43). Our results showed that L-lactate levels increased in the more severe anemia group. This is thought to be useful as an indicator of tissue oxygenation in relation to hypoxia that is induced by anemia and can also be used as an indicator of hemolytic anemia. We attempted to exclude type B hyperlactatemia by excluding other underlying diseases based on clinical symptoms.

Meanwhile, in our study, there were cattle with elevated levels of other indicators (reticulocytes count, indirect bilirubin, and L-lactate concentration) that could be identified as having hemolytic anemia, even though they were not anemic by HCT standards. This may be a case where RBC hemolysis is present but not severe, and HCT may increase due to an increase in the appearance of reticulocytes as regeneration progresses in response to hemolysis of the bone marrow. In the latter case, an increase in the MCV was observed. It may also be that hematological values were overestimated due to dehydration (44), which is commonly observed in sick cattle. Thus, it was confirmed that there may be limitations in identifying cattle with hemolytic anemia using only hematological indicators.

Cattle were classified based on HCT according to the standard of previous studies, and additional parameters with high correlation (reticulocyte count, indirect bilirubin, and L-lactate concentration) were selected to identify cattle with subclinical or insidious hemolytic anemia. Therefore, cattle with decreased HCT levels or increased additional indicators were reclassified to obtain a 95% confidence interval for the new standard anemic cattle population (Table 2). Therefore, we suggest additional criteria (values of reticulocyte count ≥1.3 × 10^3^/μL, indirect bilirubin ≥0.07 mg/dL, and L-lactate ≥1.6 mmol/L) to be used along with hematological values as indicators of bovine hemolytic anemia. Except for the reticulocyte count, the values were similar to the previously reported reference range. The reference ranges were 0 × 10^3^/μL for reticulocyte count (8, 31), 0.6–2.2 or 0.6–1.3 mmol/L for lactate concentration (45, 46), and 0–0.29 mg/dL for indirect bilirubin concentration (47).

Even if the hematological test results are normal in a situation where hemolytic anemia is suspected, the patient may be considered to have hemolytic anemia if the level rises above the additional indicator suggested. It seems possible to take preemptive measures, such as movement restrictions, quarantine, and treatment, until test results to determine the cause of hemolytic anemia, such as molecular diagnosis or toxicological tests, are available. Even in cases of asymptomatic or mild and nonspecific clinical symptoms, hemolytic anemia may be confirmed using additional indicators. Additionally, when RBCs are destroyed by hemolysis, the amount of hemoglobin that can supply oxygen decreases, resulting in hypoxia in the peripheral tissues. To compensate for the decrease in oxygenation, cardiac output increases, and continued increases in cardiac output can cause high-output cardiac failure (48, 49). Hemolytic anemia treatment can be divided into the removal of the cause of hemolysis and supportive treatment of hypoxia caused by anemia. In particular, oxygen supply and blood transfusion can be considered supportive treatments for hypoxia caused by anemia. There are various blood types in cattle, and complications may occur due to transfusion incompatibility; therefore, caution is required when transfusing (50, 51). Therefore, it is believed that classification of the severity of anemia and setting a panic value are important processes that can prevent conditions such as high-output cardiac failure caused by anemia through the application of supportive treatments such as blood transfusion and complications.

The results of this study showed that cattle with more severe hemolytic anemia tended to have increased reticulocyte count, indirect bilirubin, and L-lactate levels, which appear to reflect the severity of the hemolytic anemia. However, because the population size is small, it is insufficient to establish standards for detailed severity indicators, and because continuous evaluation has not been conducted, the prognostic relevance of the indicators is unknown. In dogs, systems called CHAOS (Canine Hemolytic Anemic Objective Score) and Tokyo score have been developed and can be used to determine the prognosis of hemolytic anemia, especially immune-mediated hemolytic anemia (52, 53). However, these systems have not been evaluated for bovine hemolytic anemia. For these reasons, additional studies are needed to establish criteria for classifying the severity of hemolytic anemia and to assess the prognostic relevance of clinical and laboratory findings.

Hematological indices in cattle can be influenced by breed, age, sex, physiological status, or environmental factors (1, 8, 31). For example, beef breeds like Hanwoo typically have higher RBC counts compared to dairy breeds, bulls have higher RBC counts than cows, and non-lactating cows have higher RBC counts than lactating cows (8, 31, 54). In young calves, RBC counts might be higher, and MCV and MCHC might be lower than in adults (8, 55). Although studies on the predisposition to hemolytic anemia in cattle are limited, it seems that the incidence of hemolytic anemia can show differences based on factors such as breed, age, and sex due to various reasons (56–60). In this study, 60 cattle were grazing and 15 were housed indoors. When anemia was classified based on HCT, 19 of the grazing cattle were classified as anemic, while none of the housed cattle were classified as anemic. However, when HCT and additional criteria were used, 55 grazing cattle and 3 housed cattle were newly classified as anemic. The higher prevalence of anemia in grazing cattle may be attributed to their increased exposure to tick-borne pathogens (18, 19). This indicates that differences in management practices, such as grazing or housing indoors, can significantly influence the hematological indices of cattle. Because the cattle selected in this study were female Korean indigenous cattle aged 22–46 months raised on the same farm, there are limitations in generalizing these findings to other conditions.

In this study, we investigated the correlation between hematological indicators and additional parameters such as reticulocyte count, indirect bilirubin, and L-lactate in Korean indigenous cattle with hemolytic anemia. A negative correlation was found between HCT and these indicators. That is, if the severity of anemia is classified based on HCT, these indicators may reflect its extent. Using these indicators, it is possible to identify cattle with hemolytic anemia that could not be detected through HCT alone. This provides a more detailed understanding of hemolytic anemia in cattle, enabling early detection and differentiation of benign cases. Ultimately, this contributes to improved animal health and farm productivity.

## Data Availability

The raw data supporting the conclusions of this article will be made available by the authors, without undue reservation.
